# Involvement of resistant bacteria in the severity of refractory osteonecrosis of the jaw

**DOI:** 10.1007/s00784-025-06547-3

**Published:** 2025-09-16

**Authors:** Junya Kusumoto, Yumi Muraki, Eiji Iwata, Megumi Matsumura, Shungo Furudoi, Masaya Akashi

**Affiliations:** 1 Department of Oral and Maxillofacial Surgery, Kobe University Graduate School of Medicine, Kobe, Japan; 2 Department of Maxillofacial Diagnostic and Surgical Science, Field of Oral and Maxillofacial Rehabilitation, Graduate school of Medical and Dental Sciences, Kagoshima University, Kagoshima, Japan; 3 Department of Oral and Maxillofacial Surgery, Faculty of Medicine, Dentistry and Pharmaceutical Sciences, Okayama University, Okayama, Japan; 4 Department of Oral Surgery, Konan Medical Center, Kobe, Japan

**Keywords:** Osteoradionecrosis of the jaw, Medication-related osteonecrosis of the jaw, Microorganism, Antimicrobial resistance, Gram-negative rod, Ampicillin

## Abstract

**Purpose:**

Osteoradionecrosis (ORN) and medication-related osteonecrosis of the jaw (MRONJ) are relatively rare and refractory, and there is no consensus regarding the bacteria associated with their development. This study was conducted to identify the bacteria associated with refractory ORN and MRONJ, including severe cases.

**Methods:**

Patients who underwent surgery for osteonecrosis of the jaw were included in this study. Bacterial culture specimens were obtained from tissue as deeply as possible. Severe cases of ORN and MRONJ were defined as stage IV of Lyon’s classification and stage III of the AAOMS classification, respectively. Demographic data, clinical features, antimicrobials usage, and bacteria detected were analysed to determine the factors associated with severe disease.

**Results:**

Seventy-seven patients (ORN, *n* = 22; MRONJ, *n* = 55) were analysed. Penicillins were the most commonly used antimicrobials. A total of 311 bacterial strains were detected in tissue culture (detection rate = 100%). *Streptococcus* spp. were the most common bacteria (37.0%), followed by anaerobes (33.8%). Gram-negative rods were detected in 10.3% of the patients, antimicrobial-resistant bacteria in 78.4%, and ampicillin resistance in 60.8%. Factors associated with severe disease were ampicillin resistance and malignancy in MRONJ, with odds ratios of 8.74 (95% confidence interval, 1.20–63.4; *p* = 0.032) and 13.5 (1.09–168, *p* = 0.043), respectively. *Enterobacter* spp. were detected only in severe cases.

**Conclusion:**

Bacteria associated with osteonecrosis of the jaw are similar in composition to those responsible for common odontogenic infections, but with a higher proportion of gram-negative rods. Ampicillin-resistant bacteria, including *Enterobacter* spp., are implicated in severe disease.

**Supplementary Information:**

The online version contains supplementary material available at 10.1007/s00784-025-06547-3.

## Introduction

Osteonecrosis of the jaw typically follows a chronic course and is often refractory to treatment, especially when managed conservatively. Osteoradionecrosis (ORN) and medication-related osteonecrosis of the jaw (MRONJ) are types of osteonecrosis that affect the jaw; however, neither occurs with high frequency, with their reported prevalence rates ranging from 5.4 to 13.2% [1] and 0.58 to 7.21% [2], respectively. The most current accepted mechanism for the pathogenesis of ORN is radiation-induced fibrosis of the tissue; that is, the radiation-induced fibroatrophic theory [3]. MRONJ is believed to be caused by the inhibition of bone remodelling through the suppression of osteoclasts [4]; however, the exact mechanism underlying its development remains unclear. Although the pathogeneses of ORN and MRONJ are believed to be different, they have similar clinical and histological features [5, 6] and are both refractory to antibacterial treatment.

Some studies have indicated that *Actinomyces* spp [7]. and common odontogenic infection-causing organisms are involved in the development of osteonecrosis of the jaw [8]. However, the methods used for specimen analysis in these studies were not consistent; some samples were assessed histopathologically [7], some were collected using swabs [9], and some were collected from tissues [10]. Collection of samples from the mouth for identification of causative organisms is associated with a high risk of contamination owing to the presence of indigenous bacteria in the oral cavity. Therefore, the optimal methods for collection of specimens from the oral cavity remain controversial. Notably, although there have been reports on specimen collection from the oral cavity in otolaryngology cases [11], no case of such specimen collection for oral maxillofacial surgery has been reported.

Narrow-range penicillins are the first choice antimicrobials for odontogenic infections. Clindamycin (CLDM) is also commonly used for the treatment of odontogenic infections [12]. However, β-lactamase-producing and CLDM-resistant bacteria have been frequently detected in osteonecrosis of the jaw [13, 14]. Moreover, osteonecrosis of the jaw may require frequent or long-term antimicrobial treatment [15], and information on the target bacteria is essential for effective empirical therapy. However, there is no consensus on the bacterial species associated with osteonecrosis of the jaw.

Factors that contribute to the severity of osteonecrosis of the jaw include age, hypoalbuminaemia, amount of exposure, and diabetes mellitus [16–19]. However, no study has been conducted to analyse the association between the causative organism and severity of osteonecrosis of the jaw. Therefore, the aim of this study was to analyse bacteria in tissue samples from patients with osteonecrosis of the jaw and determine the association between the severity of osteonecrosis of the jaw and the identified bacteria species, including resistant bacteria.

## Materials and methods

### Study design and patients

This was a retrospective case-control study of patients who underwent surgery for ORN or MRONJ at the Department of Oral and Maxillofacial Surgery, Kobe University Hospital between January 2016 and December 2023.

ORN was defined according to the Common Terminology Criteria for Adverse Events v5.0 (CTCAE ver. 5) [20]. The diagnostic criteria for ORN were devitalisation and exposure of bone through the overlying mucosa within the irradiated area (grade ≥ 2, CTCAE ver. 5) that persisted without healing for three months in the absence of tumour recurrence [21, 22]. MRONJ was diagnosed based on the following diagnostic criteria proposed by the American Association of Oral and Maxillofacial Surgeons (AAOMS) [23]: (1) previous or current treatment with anti-resorptive agents (ARAs) alone or in combination with immunomodulatory or angiogenesis inhibitors, (2) bone exposed through an intraoral or extraoral fistula in the maxillofacial region lasting for more than 8 weeks or capable of exploration, and (3) no history of radiation therapy to the jaws or disease metastasis to the jaws. The inclusion criterion was tissue culture of surgical specimens.

Specimens were collected from the deepest part of the lesion. In addition, the specimens were collected from multiple tissues (necrotic bone, granulation tissue, periosteal reaction, and pus) if possible. Pus samples were collected using sampling swabs. The tissues were stored in sterile spits filled with saline solution immediately after collection. Samples from different types of tissues were stored separately. The exclusion criteria were missing laboratory data and unavailable data on antimicrobial usage.

## Data collection

Patient medical data were extracted from electronic medical records. The data collated for analysis included patient characteristics (age, sex, body mass index, smoking and drinking history, immunocompromised status [diabetes mellitus, corticosteroid usage], cancer-bearing status), data on haematological examination performed just before surgery (albumin [g/dL], haemoglobin [g/dL], estimated glomerular filtration rate [mL/min/1.73 m^2^], white blood cell count [/µL] and its fractions [number of neutrophils and lymphocytes]), osteonecrosis characteristics (site [maxilla, mandible], type of osteonecrosis [ORN, MRONJ], stage classification, history of cellulitis), the period from diagnosis of osteonecrosis of the jaw to surgery, presence and frequency of antimicrobial use before surgery, duration of antimicrobial use (oral, intravenous) before surgery, and bacteria detected in tissue culture. For patients with MRONJ, we also investigated whether ARAs were administered for osteoporosis or malignancy.

The severity of ORN was categorised according to Lyon’s classification [24]: stage I, affected (damaged or exposed) bone < 2.5 cm; stage II, affected bone > 2.5 cm, asymptomatic; stage III, affected bone > 2.5 cm, symptomatic; and stage IV, affected bone with pathological fracture, orocutaneous fistula, or involvement of the inferior alveolar nerve. The severity of MRONJ was categorised using the AAOMS stage classification [23]: stage I, exposed and necrotic bone or fistula that probes to the bone in patients who are asymptomatic and have no evidence of infection/inflammation; stage II, exposed and necrotic bone or fistula that probes to the bone, with evidence of infection/inflammation; and stage III, exposed and necrotic bone or fistulae that probe to the bone, with evidence of infection, and one or more of the following: exposed necrotic bone extending beyond the region of alveolar bone (i.e., inferior border and ramus in the mandible, maxillary sinus, and zygoma in the maxilla), pathologic fracture, extraoral fistula, oral antral/oral-nasal communication, and osteolysis extending to the inferior border of the mandible or sinus floor. Severe cases were defined as stage IV for ORN and stage III for MRONJ.

Our institution has been accredited to ISO 15,189 (certification number, RML00980), with standard operating procedures established and implemented for all inspection processes. Following the methods outlined in a previous report [25], antimicrobial susceptibility testing was performed using microbiological laboratory records according to the Clinical and Laboratory Standards Institute recommendations.

## Endpoints

The primary endpoint was confirmation of the relationship between the severity of osteonecrosis of the jaw and bacterial species, including resistant bacteria. The secondary endpoint was identification of the bacteria associated with osteonecrosis of the jaw and confirmation of potential differences in the bacteria associated with ORN and MRONJ. The third endpoint was identification of other factors associated with the severity of osteonecrosis of the jaw.

### Statistical analysis

Representative values are presented as medians with the first and third quartiles. Fisher’s exact test was used for comparisons of nominal variables across disease stages. The Brunner–Munzel test was used for two-group comparisons of continuous variables. The variables associated with disease severity in the univariate analysis were included in a multivariate logistic regression model. All statistical analyses were performed using the R software version 4.1.0 (R Development Core Team, 2021; R Foundation for Statistical Computing, Austria). The significance level was set at *p* < 0.05.

## Results

### Patient and osteonecrosis characteristics

**Fig. 1:**
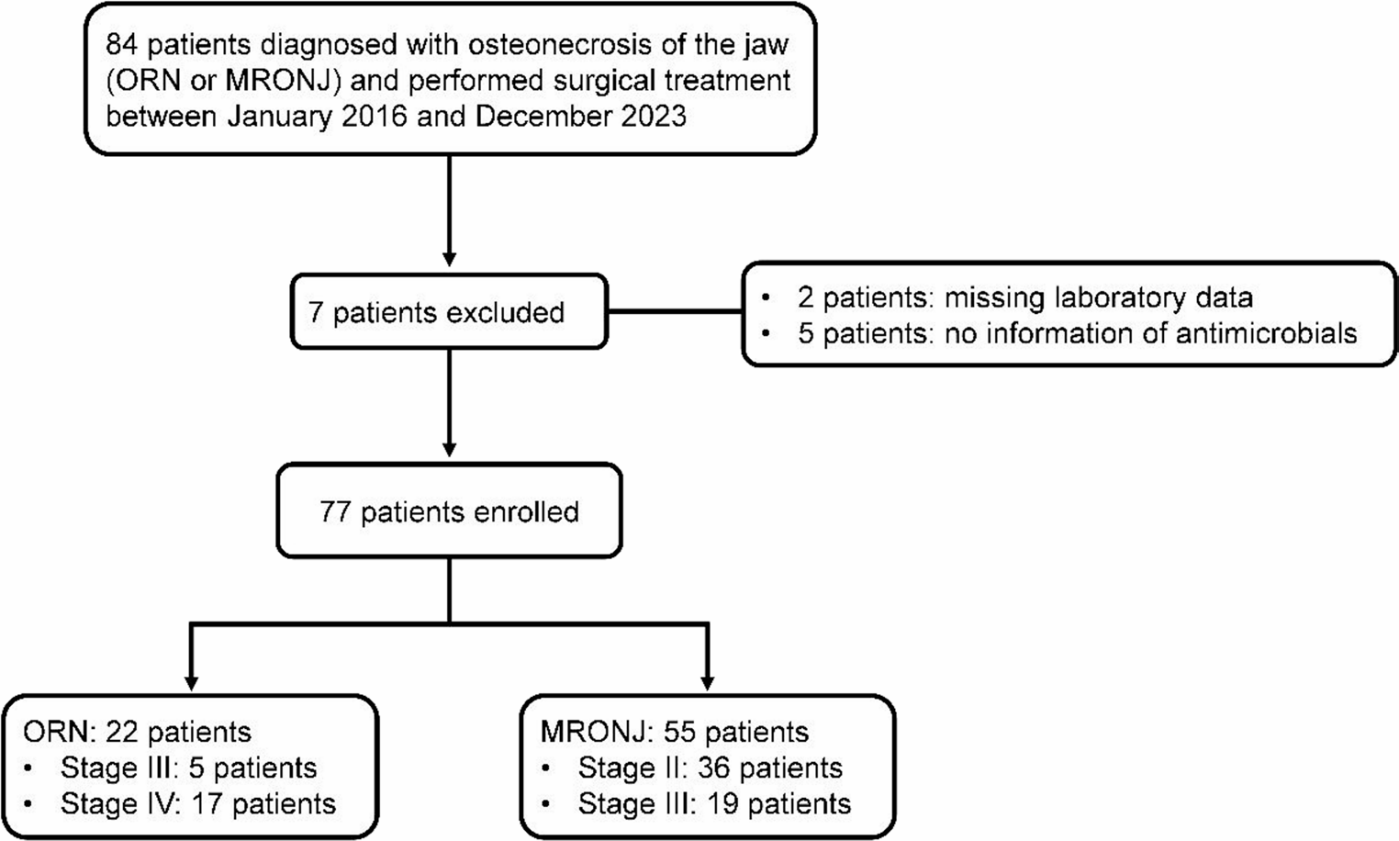
Flowchart of data collection and cleaning MRONJ, medication-related osteonecrosis of the jaw; ORN, osteoradionecrosis of the jaw

Of 84 patients who were diagnosed with ORN or MRONJ and underwent surgical treatment, 77 patients met the inclusion criteria and seven were excluded (Fig. 1). Of the 77 patients, 22 had ORN and 55 had MRONJ. Approximately half of the patients were immunosuppressed. Osteonecrosis of the jaw occurred in the mandible in most cases. All the 22 patients with ORN (28.6%) received radiation therapy of 60 Gy or higher (local radiation dose unknown). Of the 55 patients with MRONJ (71.4%), 33 developed the disease due to use of osteoporosis medications, whereas 22 developed the disease due to treatment for malignant tumours. Of the 77 patients in the entire cohort, 36 (46.8%) had severe disease. In addition, approximately half of the patients had a history of cellulitis. The median period from diagnosis of osteonecrosis of the jaw to surgery was 136 days. Most of the patients were treated with antimicrobials, which were administrated intravenously in half of the cases (Table 1).

**Table 1:** Characteristics of the patients

Variables	Number of patients (*n* = 77)
Patient characteristics	
Age (years)	73 (68, 82)
Sex (male)	27 (35.1%)
Body mass index	20.9 (19.1, 23.4)
History of smoking	15 (19.5%)
Alcohol consumption	20 (26.0%)
Immunocompromise	43 (55.8%)
Cancer-bearing	23 (29.9%)
Haematologic examination	
Albumin (g/dL)	3.8 (1.6, 4.9)
Haemoglobin (g/dL)	11.6 (6.3, 15.6)
eGFR (mL/min/1.73m^2^)	59.9 (46.6, 72.3)
White blood cell (/µL)	6250 (2400, 14400)
Neutrophil (%)	66 (25, 92)
Cell count	4019 (2989, 5398)
Lymphocyte (%)	23 (3, 54)
Cell count	1402 (903, 1818)
Osteonecrosis characteristics	
Location	
Maxilla	13 (16.9%)
Mandible	64 (83.1%)
Type of osteonecrosis	
ORN	22 (28.6%)
MRONJ	55 (71.4%)
Underlying osteoporosis	33 (60.0%)
Underlying malignancy	22 (40.0%)
ORN stage	
Stage III	5 (22.7%)
Stage IV	17 (77.3%)
MRONJ stage	
Stage II	36 (65.5%)
Stage III	19 (34.5%)
Severe condition	36 (46.8%)
Cellulitis	38 (49.4%)
Duration from diagnosis to surgery (days)	136 (61, 441)
Use of antimicrobials before surgery	68 (88.3%)
Intravenous administration	34 (44.2%)

Data are presented as median (first quartile, third quartile) or n (%)

## Characteristics of antimicrobials

**Fig. 2:**
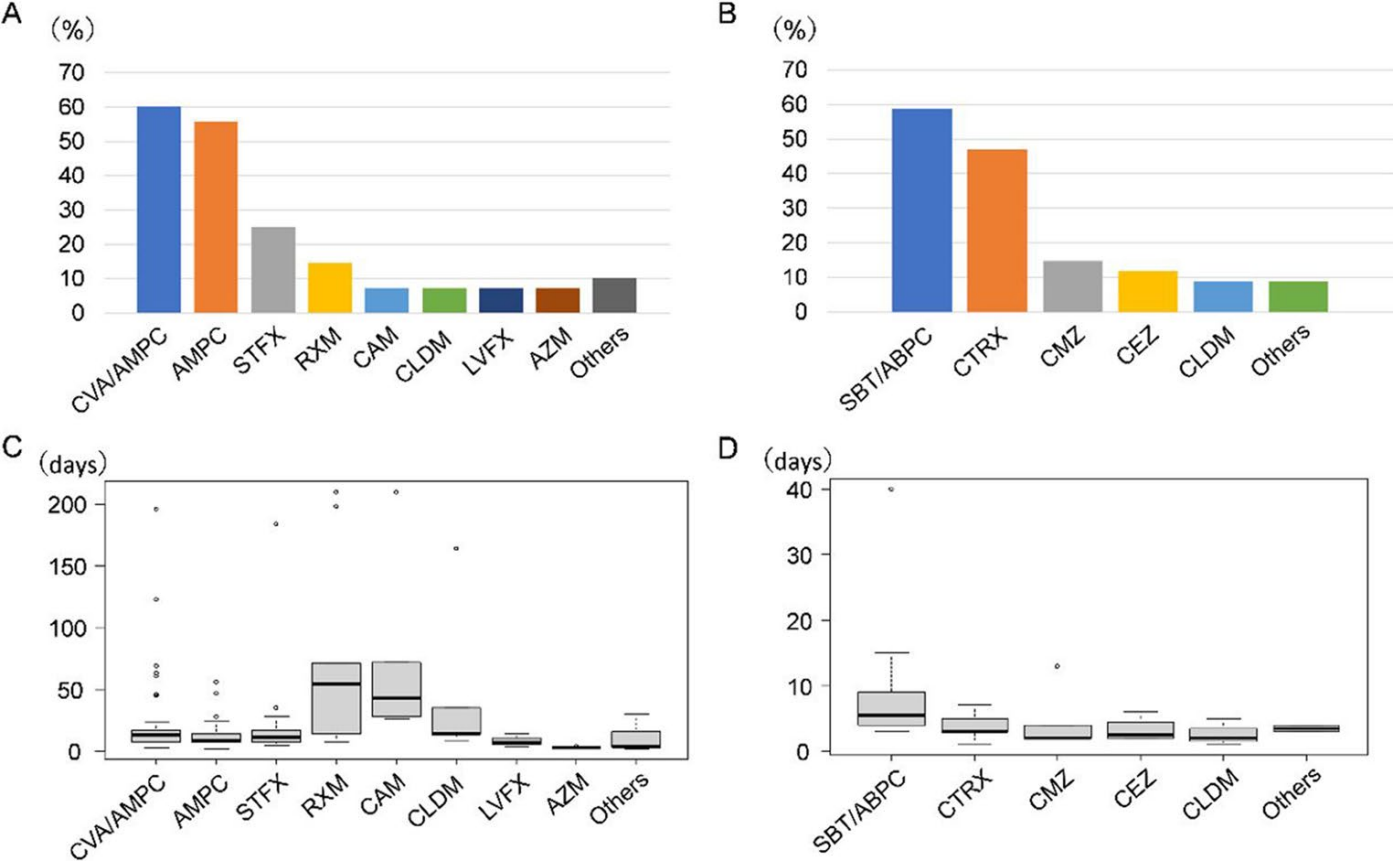
Types and quantity of antimicrobials used before surgery (**A**) Usage rate for oral antimicrobials. (**B**) Usage rate for intravenous antimicrobials. (**C**) Duration of use (number of days) for each oral antimicrobial. (**D**) Duration of use (number of days) for each intravenous antimicrobial AMPC, amoxicillin; AZM, azithromycine; CAM, clarithromycine; CEZ, cefazorin; CLDM, clindamycine; CMZ, cefmetazole; CTRX, ceftriaxon; CVA/AMPC, clavulanic acid/amoxicillin; LVFX, levofloxacin; RXM, roxithromycine; SBT/ABPC, sulbactam/ampicillin; STFX, sitafloxacine

Antimicrobials were administered during the acute inflammatory phase and continued when symptoms persisted, even after objective signs of inflammation had disappeared. The penicillin-based antimicrobials were mostly administered orally, especially amoxicillin with clavulanic acid (CVA/AMPC), a beta-lactamase inhibitor (Fig. 2A). Sulbactum/ampicillin (SBT/ABPC), a combination penicillin-derived beta-lactamase inhibitor, was the most commonly used injectable drug, followed by ceftriaxon, a third-generation cephem antibacterial (Fig. 2B). Regarding duration of use, the median duration of use per dose was more than one month for oral macrolides (roxithromycine, RXM; clarithromycine, CAM) (Fig. 2C). SBT/ABPC was the most commonly administered intravenous antimicrobial, with a median duration of use of 6 days per dose (Fig. 2D).

## Bacteria detected in tissue culture

**Fig. 3:**
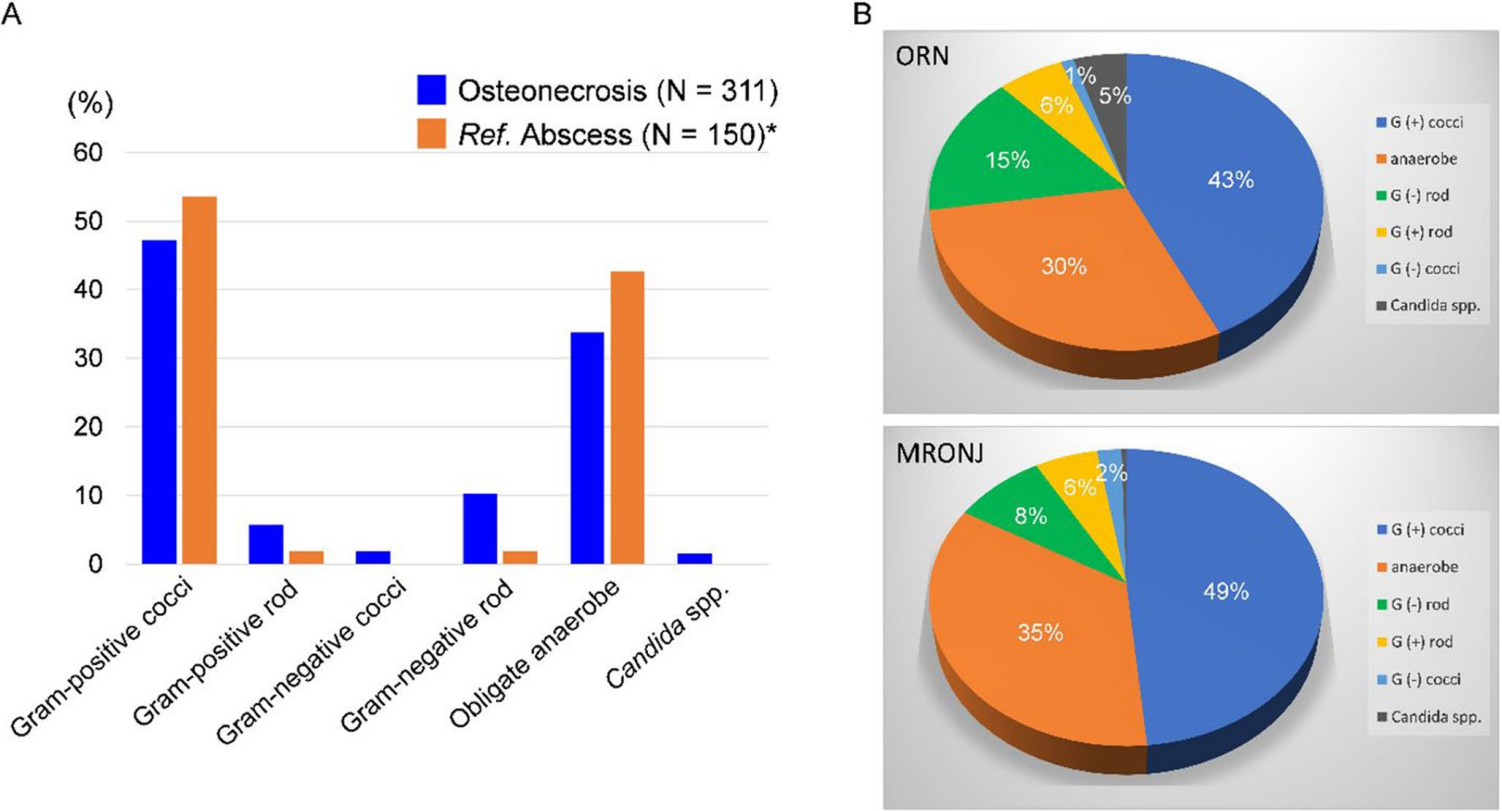
Bacterial species detected in tissue cultures (**A**) Comparison of bacteria species detected with common odontogenic infection-causing bacteria. As a reference, bacteria detected in abscesses caused by odontogenic infections other than osteomyelitis were shown (partly modified from Ref. 26). Among the gram-positive cocci, *Streptococcus* spp. were detected in 37.3%. (**B**) Percentage of bacteria detected in specimens from patients with ORN and MRONJ MRONJ, medication-related osteonecrosis of the jaw; ORN, osteoradionecrosis of the jaw

The bacterial detection rate in this study was 100%. Multiple tissue samples were collected from 39 patients (50%), and the bacteria detected in the multiple tissues were consistent for 31 patients (80%). A total of 311 bacterial strains were detected in the tissue cultures. Gram-positive cocci were the most common, accounting for 145 strains (46.6%), 78% of which were *Streptococcus* spp. (particularly *S. anginosus*). A total of 105 strains (33.8%) of obligate anaerobes were detected, 37% of which were *Prevotella* spp. Five strains of *Candida* spp. were detected. *Actinomyces* spp. were detected less frequently, with only seven isolates identified (2.3%). Notably, the composition of the bacteria detected in this study was similar to that previously reported for bacteria detected in abscesses [26]; however, a higher proportion of gram-negative rods was detected in this study (Fig. 3A; Table 2). Regarding types of osteonecrosis, the composition of bacteria detected in patients with ORN and MRONJ was almost the same; however, a higher proportion of gram-negative rods was detected in patients with ORN (Fig. 3B). There was no significant difference in the period from diagnosis of osteonecrosis of the jaw to surgery in cases where obligate anaerobes were detected (*p* = 0.992). In contrast, cases in which Gram-negative rods were detected had a significantly longer period from diagnosis of osteonecrosis to surgery (*p* = 0.037), as well as a longer duration of antimicrobial use (median 55 days vs. 18 days, *p* = 0.025).

**Table 2:** Bacteria detection rate categorised according to type of osteonecrosis

	GPC	No. (%)	GPR	No. (%)	GNC	No. (%)	GNR	No. (%)	Obligate anaerobe	No. (%)
[ORN]										
Stage III (*N* = 12)	*Streptococcus* spp.	6 (50%)	*Lactobacillus* spp.	1 (8.3%)	*Neisseria* spp.	1 (8.3%)			*Prevotella* spp.	1 (8.3%)
			*Bacillus* spp.	1 (8.3%)					Others	2 (16.7%)
Stage IV (*N* = 66)	*Streptococcus* spp.	21 (31.8%)	*Actinomyces* spp.	1 (1.5%)	-		*Enterobacter* spp.	5 (7.6%)	*Prevotella* spp.	9 (13.6%)
	*Staphylococcus* spp.	5 (7.6%)	*Lactobacillus* spp.	1 (1.5%)			*Klebsiella* spp.	2 (2.8%)	*Parvimonas micra*	2 (3.0%)
	*Enterococcus* spp.	1 (1.5%)	*Corynebacterium* spp.	1 (1.5%)			*Escherichia coli*	1 (1.5%)	*Fusobacterium* spp.	2 (3.0%)
	*Gemella* spp.	1 (1.5%)					*Pseudomonas* spp.	1 (1.5%)	*Bacteroides* spp.	1 (1.5%)
							Others	4 (6.1%)	Others	8 (12.1%)
[MRONJ]										
Stage II (*N* = 144)	*Streptococcus* spp.	57 (39.6%)	*Actinomyces* spp.	5 (3.5%)	*Neisseria* spp.	3 (2.1%)	*Eikenella* spp.	4 (2.8%)	*Prevotella* spp.	17 (11.7%)
	*Staphylococcus* spp.	7 (4.9%)	*Bacillus* spp.	2 (1.4%)			*Klebsiella* spp.	2 (1.4%)	*Parvimonas micra*	8 (5.6%)
	*Granulicatella* spp.	2 (1.4%)	*Corynebacterium* spp.	1 (0.7%)			*Escherichia coli*	1 (0.7%)	*Fusobacterium* spp.	6 (4.2%)
	*Enterococcus* spp.	1 (0.7%)					*Citrobacter* spp.	1 (0.7%)	*Bacteroides* spp.	2 (1.4%)
	Others	3 (2.1%)					*Haemophilus* spp.	1 (0.7%)	Others	21 (14.6%)
Stage III (*N* = 84)	*Streptococcus* spp.	29 (34.5%)	*Lactobacillus* spp.	2 (2.4%)	*Neisseria* spp.	2 (2.4%)	*Enterobacter* spp.	5 (6.0%)	*Prevotella* spp.	12 (14.3%)
	*Staphylococcus* spp.	6 (7.1%)	*Actinomyces* spp.	1 (1.2%)			*Escherichia coli*	2 (2.4%)	*Fusobacterium* spp.	5 (6.0%)
	*Enterococcus* spp.	2 (2.4%)	*Corynebacterium* spp.	1 (1.2%)			*Pseudomonas* spp.	1 (1.2%)	*Parvimonas micra*	2 (2.4%)
	*Granulicatella* spp.	1 (1.2%)	*Bacillus* spp.	1 (1.2%)			*Haemophilus* spp.	1 (1.2%)	*Bacteroides* spp.	1 (1.2%)
	Others	3 (3.6%)					*Rahnella* spp.	1 (1.2%)	Others	6 (7.1%)

*Candida* spp. were excluded from this table (*N* = 5, stage IV ORN 4, stage II MRONJ 1)

Abbreviations: GPC, gram-positive cocci; GPR, gram-positive rods; GNC, gram-negative cocci; GNR, gram-negative rods; MRONJ, medication-related osteonecrosis of the jaw; ORN, osteoradionecrosis of the jaw

Fifty-one patients were tested for antimicrobial susceptibility. Acquired resistant bacteria were detected in 40 patients (78.4%). The resistance rates for ABPC (AMPC), CLDM, macrolide antimicrobials, and quinolones commonly used in oral and maxillofacial surgery were investigated. The results showed that 31 (60.8%) patients were ABPC-resistant, 19 (37.4%) were CLDM-resistant, 30 (58.8%) were macrolide-resistant, and 16 (31.4%) were quinolone-resistant. A total of 197 bacterial strains were detected, 62 (31.5%) and 42 (21.3%) of which were completely resistant and ABPC-resistant strains, respectively (Table 3).

**Table 3:** Detection rates for resistant bacteria

Variables	Antimicrobial resistance	No effect of ABPC
Patients with resistant bacteria (*N* = 51)	40 (78.4%)	31 (60.8%)
Bacteria species (*N* = 197)	62 (31.5%)	42 (21.3%)
Gram positive cocci (92)	46 (50.0%)	15 (16.3%)
*Streptococcus* spp. (71)	30 (42.3%)	2 (2.8%)
*Staphylococcus* spp. (13)	11 (84.6%)	11 (84.6%)
*Enterococcus* spp. (4)	4 (100%)	2 (50.0%)
Others (4)	1 (25.0%)	0
Gram positive rods (7)	2 (28.6%)	1 (14.3%)
*Actinomyces* spp. (2)	0	0
*Corynebacterium* spp. (2)	1 (50.0%)	1 (50.0%)
Others (3)	1 (33.3%)	0
Gram negative cocci (3)	0	0
Gram negative rods (28)	5 (17.9%)	19 (67.9%)
*Enterobacter* spp. (10)	2 (20.0%)	10 (100%)
*Escherichia coli* (5)	1 (20.0%)	0
*Klebsiella* spp. (4)	0	4 (100%)
*Pseudomonas* spp. (2)	1 (50.0%)	2 (100%)
*Citrobacter* spp. (2)	0	2 (100%)
Others (5)	1 (20.0%)	1 (20%)
Obligate anaerobes (67)	9 (13.4%)	7 (10.4%)
*Prevotella* spp. (30)	6 (20.0%)	5 (16.7%)*
*Fusobacterium* spp. (10)	0	0
*Parvimonas micra* (6)	0	0
*Bacteroides* spp. (2)	2 (100%)	2 (100%)
Orthers (19)	1 (5.3%)	0

Data are shown as median (first quartile, third quartile) or n (%)

Abbreviations: ABPC, ampicillin

*Beta-lactamase production

### Factors associated with severity

Factors associated with disease severity were investigated in 51 patients who had results of susceptibility testing. Univariate analysis revealed statistically significant differences in age, type of osteonecrosis, history of cellulitis, period from diagnosis of osteonecrosis of the jaw to surgery, presence of gram-negative rods (*Enterobacter* spp.), and antimicrobial resistance (ABPC-resistant) between the severe disease (stage IV of ORN and stage III of MRONJ) and non-severe disease groups (Table 4). Therefore, a multivariate analysis was conducted using these variables as explanatory variables. The results showed statistically significant differences in MRONJ (underlying malignancy) and ABPC resistance between the two groups, with odds ratios of 13.5 (95% confidence interval [CI], 1.09–168; *p* = 0.043) and 8.74 (95% CI, 1.20–63.4; *p* = 0.032), respectively (Table 5). *Enterobacter* spp. were detected only in the severe disease group.

**Table 4:** Univariate analysis of factors that affect the severity of osteonecrosis of the jaw

Variables	Severe (*n* = 29)	Not severe (*n* = 22)	*p* value
Patient characteristics			
Age (years)	70 (66, 74)	82 (67, 86)	0.038^*^
Sex (male)	13 (44.8%)	4 (18.2%)	0.072
Body mass index	20.4 (19.2, 23.1)	20.9 (19.8, 23.2)	0.216
History of smoking	6 (20.7%)	4 (18.2%)	1.000
Alcohol consumption	10 (34.5%)	5 (22.7%)	0.536
Immunocompromised status	16 (44.8%)	12 (54.4%)	0.577
Cancer-bearing	9 (31.0%)	5 (22.7%)	0.546
Haematologic examination			
Albumin (g/dL)	3.8 (3.3, 4.1)	3.7 (3.4, 3.9)	0.793
Haemoglobin (g/dL)	12.0 (10.4, 13.1)	11.3 (10.3, 13.2)	0.620
eGFR (mL/min/1.73m^2^)	63.8 (57.7, 78.7)	58.8 (46.3, 72.6)	0.221
White blood cell (/µL)	5550 (4500, 7100)	5900 (5100, 8300)	0.515
Neutrophil (/µL)	3900 (2959, 5043)	4213 (2968, 5760)	0.613
Lymphocyte (/µL)	1160 (896, 1592)	1475 (1263, 1718)	0.275
Osteonecrosis characteristics			
Location			0.728
Maxilla	5 (17.2%)	5 (22.7%)	
Mandible	24 (82.8%)	17 (77.3%)	
Type of osteonecrosis			0.010^*^
ORN	13 (44.8%)	4 (18.2%)	
MRONJ			
For osteoporosis	6 (20.7%)	14 (63.6%)	
For malignancy	10 (34.5%)	4 (18.2%)	
Cellulitis	21 (72.4%)	8 (36.4%)	0.021^*^
Duration from diagnosis to surgery (days)	324 (77, 823)	90 (46, 344)	0.018^*^
Bacteria characteristics			
Gram-negative rod	17 (58.6%)	5 (22.7%)	0.013^*^
*Enterobacter* spp.	10 (34.5%)	0	0.003^*^
Others	7 (24.1%)	5 (22.7%)	1.000
Antimicrobial resistance			
ABPC	23 (79.3%)	8 (36.4%)	0.003^*^
CLDM	9 (31.0%)	10 (45.5%)	0.383
Macrolide	15 (51.7%)	15 (68.2%)	0.266
Quinolone	9 (31.0%)	7 (31.8%)	1.000

Data are shown as median (first quartile, third quartile) or n (%)

^*^ Statistically significant (*p* < 0.05)

ABPC, ampicillin; CLDM, clindamycin; eGFR, estimated glomerular filtration rate; MRONJ, medication-related osteonecrosis of the jaw; ORN, osteoradionecrosis of the jaw

**Table 5:** Multivariate logistic regression analysis of factors that affect the severity of osteonecrosis of the jaw

Variables	β	OR	95% CI		*p* value
			Lower	Upper	
Age	-0.04	0.96	0.87	1.05	0.389
Cellulitis	0.94	2.57	0.40	16.7	0.322
Type of osteonecrosis (Ref. MRONJ [osteoporosis])					
MRONJ (malignancy)	2.61	13.5	1.09	168	0.043^*^
ORN	1.32	3.76	0.31	45.8	0.299
Duration from diagnosis to surgery	< 0.01	1.00	1.00	1.00	0.508
Gram-negative rods	1.01	2.75	0.39	19.4	0.310
ABPC resistance	2.17	8.74	1.20	63.4	0.032^*^

^*^ Statistically significant (*p* < 0.05)

Abbreviations: ABPC, ampicillin; CI, confidence interval; MRONJ, medication-related osteonecrosis of the jaw; OR, odds ratio; ORN, osteoradionecrosis

## Discussion

In this study, we analysed tissue cultures of bacteria associated with osteonecrosis of the jaw and evaluated the association between the identified bacteria species and the severity of osteonecrosis of the jaw. The results of this study indicated that the bacteria associated with osteonecrosis of the jaw have a similar composition to that of bacteria associated with common odontogenic infections, but a higher proportion of gram-negative rods (especially in samples from patients with ORN). In addition, we observed that *Enterobacter* spp. were detected only in severe cases. Furthermore, the severity of osteonecrosis of the jaw was associated with the presence of ABPC-resistant bacteria (an approximately 9-fold increased risk) and the type of osteonecrosis of the jaw (underlying malignancy in MRONJ) (an approximately 14-fold increased risk). To our knowledge, the present study is the first study conducted to examine the association between bacteria and the severity of osteonecrosis of the jaw.

The oral cavity has endemic flora, and contamination during bacteriological examinations is unavoidable. Therefore, it is important to reduce the risk of contamination to ensure accurate identification of causative agents. Current guidelines indicate that in the management of diabetic foot lesions, which is a leading chronic infectious disease, the specimens should be taken from tissue as deeply as possible for bacterial testing before initiation of antimicrobial therapy [27]. In addition, it is recommended that tissue culture be performed in such cases instead of swab testing [28]. As we considered that bacterial testing should also be adopted for osteonecrosis of the jaw, the specimens in this study were obtained from tissue as deeply as possible.

In this study, oral *Streptococcus* spp. and obligate anaerobes were the bacteria predominantly detected in tissue cultures, with a composition similar to that observed in common odontogenic infections [13, 26, 29]; however, a higher proportion of gram-negative rods was detected in the present study. The bacteria detected in the present study are similar to those reported in previous studies [10, 14, 30, 31]. Among the gram-negative rods, *Enterobacter* spp. were most frequently detected in the present study. Notably, similar findings have been reported in previous studies [32]. The high proportion of gram-negative rods detected in the present study could be attributed to a long-term medical history of treatment of the primary disease and microbial substitution due to long-term use of antimicrobials [31]. However, narrow-range penicillins such as ABPC are typically the first choice for odontogenic infections because *Streptococcus* spp. are the primary causative organisms in such cases [12]. As *Streptococcus* spp. were also detected in most specimens in the present study, penicillins were the first choice of treatment for the patients. However, the detection rate for ABPC-resistant bacteria was as high as approximately 60%, which is comparable to that reported in a previous study [31]. In addition, *Enterobacter* spp. and *Klebsiella* spp., which were detected in the present study, are naturally resistant to penicillins. Moreover, many gram-negative rods (e.g. *Pseudomonas* spp.) are not in the ABPC spectrum. Therefore, if antimicrobial treatment is necessary, empirical treatment should be started with a penicillin-based antimicrobial combined with a beta-lactamase inhibitor; thereafter, the drug can be changed to a suitable antimicrobial based on the bacterial culture results.

Factors associated with the severity of osteonecrosis of the jaw were examined in this study, and the results suggested that ABPC-resistant bacteria are associated with osteonecrosis of the jaw. Necrotic bones have poor blood flow and are difficult to treat using antimicrobials. Therefore, it is necessary to physically remove the necrotic tissue to reduce the quantity of the bacteria present. However, complete debridement is often difficult if the patient’s general condition and the boundaries of the necrotic bone are unclear. Therefore, conservative treatments such as washing and administering antimicrobials are often used in clinical practice [33]. Frequent use of the oral antimicrobials CVA/AMPC and AMPC was observed in the present study. However, macrolides and quinolones may have been used in a diffuse manner. Therefore, accumulation of resistant bacteria in the necrotic bone and difficulty achieving complete debridement may have contributed to the spread of the lesions. This indicates that it is important to remove necrotic bone whenever possible and encourage the appropriate use of antimicrobials, even in cases of osteonecrosis of the jaw [34]. In a recent study, aggressive surgical treatment yielded good results, particularly for patients with MRONJ [35].

Factors associated with severe ORN have been reported previously. These factors include age, irradiation dose, postoperative radiation, diabetes, heavy smoking, and heavy drinking [16, 17]. Notably, none of these factors were identified in the present study. The factors associated with MRONJ include younger age, hypoalbuminaemia (< 4.0 g/dL), maxillary onset, and chemotherapy (cancer-bearing) [18, 19]. In addition, failure to cure has been reported in patients with underlying disease (cancer) [19]. The results of the present study indicated that MRONJ resulting from the treatment of a malignancy is a critical factor associated with the severity of osteonecrosis of the jaw, a finding that is consistent with the results of previous studies. However, the wide range of the 95% CIs indicates that the results should be interpreted with caution. In this study, the number of surgically treated MRONJ cases was lower in patients with malignancy-related disease than in those with osteoporosis-related disease. This may be attributed to the poor general condition and limited prognosis of patients with malignancy, which often precludes surgical intervention.

This study has some limitations. First, the retrospective design of the study may have introduced observer and recorder bias into the data collection process. To reduce this bias, the data were collected and recorded by three independent observers (JK, MY, and MM). Second, as the risk of specimen contamination could not be completely eliminated, the true relevance of the detected bacteria remains unclear. This is the greatest limitation in the analysis of specimens obtained from the oral cavity. To combat this issue, samples were collected from multiple tissues as deeply as possible, thereby improving the reliability of the detected bacteria being the causative organism. Third, susceptibility testing was not performed in all cases. This means that susceptibility testing may have been actively conducted for bacteria with a high likelihood of becoming resistant; therefore, the risk of selection bias cannot be completely ruled out. Although the dosage and duration of antibiotic use may contribute to the development of resistant bacteria, this study did not investigate these factors, as they could not be determined from the current data alone. This remains an important area for future research. Fourth, all the patients included in this study underwent surgical treatment. Therefore, whether the results of this study can be directly applied to patients who have not undergone surgery is unclear. Nevertheless, it is advisable to actively perform tissue culture at any stage of osteonecrosis of the jaw to ensure the appropriate use of antimicrobials. As this was a retrospective study, future prospective studies with sufficient sample sizes are needed to validate the findings.

## Conclusions

This study demonstrated that common odontogenic pathogenic bacteria such as *Streptococcus* spp. and obligate anaerobes are associated with both ORN and MRONJ. Notably, gram-negative rods, particularly *Enterobacter* spp., were detected more frequently only in severe cases. The results of this study suggest that ABPC-resistant bacteria are involved in severe osteonecrosis of the jaw. Furthermore, the results indicate that MRONJ caused by the use of ARAs for the treatment of malignant tumours have a high likelihood of being severe.

**Statements and Declarations**.

## Supplementary Information

Below is the link to the electronic supplementary material.

Supplementary Material 1

## Data Availability

The datasets used and analysed in the current study are available from the corresponding author upon reasonable request.

## Abbreviations

eGFR: estimated glomerular filtration rate
MRONJ: medication-related osteonecrosis of the jaw
ORN: osteoradionecrosis of the jaw
