# Supplementary material for: Involvement of resistant bacteria in the severity of refractory osteonecrosis of the jaw
Source: Clin Oral Investig. 2025 Sep 16;29(10):454. doi: 10.1007/s00784-025-06547-3 (PMC12441049; doi:10.1007/s00784-025-06547-3)
Supplement: Supplementary file 1 — Supplementary Material 1 [file 784_2025_6547_MOESM1_ESM.docx]

**Supplementary Table 1. Detection of bacteria from tissue culture**

| Variables | N = 77 | concordance |
| --- | --- | --- |
| Detection from tissue culture | 77 (100%) |  |
| Multiple tissue collection | 39 (50%) | 31 (80%) |
| Necrotic bone–granulation tissue | 28 | 21 (75%) |
| Necrotic bone–granulation tissue–periosteum | 2 | 1 (50%) |
| Necrotic bone–periosteum | 1 | 1 (100%) |
| Necrotic bone–pus | 2 | 2 (100%) |
| Granulation tissue–pus | 6 | 6 (100%) |
| Number of bacteria detected per case | 4 (3, 5) |  |

Data are shown as the median (first quartile, third quartile) or n (%).
